# DEVELOPING A RESOURCE FOR LEVERAGING POPULATION DATA SETS TO ADVANCE CAREGIVING SCIENCE

**DOI:** 10.1093/geroni/igac059.2110

**Published:** 2022-12-20

**Authors:** Rebecca Goodwin, Katherine Ornstein, Catherine Elmore, Rebecca Utz, Djin Tay, Lee Ellington, Ken Smith, Caroline Stephens

**Affiliations:** University of Utah, Salt Lake City, Utah, United States; Johns Hopkins, University, Baltimore, Maryland, United States; University of Utah, Salt Lake City, Utah, United States; University of Utah, Salt Lake City, Utah, United States; University of Utah, Salt Lake City, Utah, United States; University of Utah and Huntsman Cancer Institute, Salt Lake City, Utah, United States; University of Utah, Salt Lake City, Utah, United States; University of Utah, Salt Lake City, Utah, United States

## Abstract

Older adults often rely on other people to help care for them as they age. Caregiving has predominantly been studied through a primary caregiver lens with few studies focusing on the wider caregiving network. However, it is challenging to collect new data about family caregivers, particularly in contexts of serious illness and end of life. Selection and attrition effects, underrepresentation of marginalized or minoritized racial and ethnic groups, and low response rates can bias findings in prospective clinical research. Analyzing existing datasets, particularly population-based data (e.g., registries with linked administrative, health, and vital records) may hold promise to advance our understanding of the role families play throughout the caregiving and bereavement continuum to optimize the health and well-being of older adults. Little is known, however, about existing population-based datasets available for research on family caregiving. We conducted a scoping review of published literature and data repositories to identify datasets relevant for family caregiving research, explore the research reuse of these datasets, and describe potential validity and reliability concerns. Synthesized findings reveal: 1) methodological approaches for identifying the presence and type of caregivers and their level of engagement; 2) inclusion and measurement of key outcome variables; and 3) sampling and study design issues. We describe and compare a selection of high-value existing datasets relevant to family caregiving research. This new research information resource will advance research use of population datasets to improve care for older adults and their family caregivers across the lifespan.

